# Possible spontaneous regression of hepatocellular carcinoma with unique histopathological features confirmed by surgical resection: a case report

**DOI:** 10.1186/s40792-021-01246-z

**Published:** 2021-07-13

**Authors:** Toshihisa Kimura, Takanori Goi, Shigehiro Yokoi, Kenji Ohnishi, Tamotsu Togawa, Atsushi Iida, Makoto Ishida, Yasunori Sato

**Affiliations:** 1 Department of Surgery, National Hospital Organization, Tsuruga Medical Center, 33-1, Sakuragaoka, Tsuruga, Fukui 914-0195 Japan; 2 First Department of Surgery, Faculty of Medicine, University of Fukui, 23-3, Matsuoka Shimoaizuki, Eiheiji-cho, Yoshida-gun, Fukui 910-1193 Japan; 3 Department of Surgery, Tannan Regional Medical Center, 1-2-31, Saburoku-cho, Sabae, Fukui 916-8515 Japan; 4 Department of Human Pathology, Kanazawa University Graduate School of Medicine, 13-1, Takara-machi, Kanazawa, Ishikawa 920-8640 Japan

**Keywords:** Hepatocellular carcinoma, Spontaneous regression, Immune response, Tumor-infiltrating lymphocyte

## Abstract

**Background:**

Spontaneous regression of hepatocellular carcinoma (HCC) is a rare event, and its clinicopathological features and underlying mechanism are not fully understood.

**Case presentation:**

An 84-year-old female with hepatitis C virus infection and diabetes mellitus was referred to our hospital for further examination. Abdominal ultrasonography showed a 3.4-cm solid tumor with a heterogeneous irregular center and no fibrous capsule in liver segment 8 (S8). An enhanced computed tomography (CT) scan revealed a tumor in S8 with heterogeneous enhancement in the arterial phase and washed out insufficiently in the portal and equilibrium phase. The enhanced pattern on magnetic resonance imaging was similar to that of CT. Although the imaging findings were not typical for HCC, liver resection (S8) was performed with HCC as the most probable diagnosis. Histopathological examination of the resected specimen showed that the tumor was well to moderately differentiated HCC with unique features. Approximately half of the tumor was composed of well-differentiated HCC that was focally accompanied by dense lymphocyte infiltration. The other half of the tumor was fibrotic tissue that resembled an inflammatory pseudotumor. Several foci of moderately differentiated HCC were scattered within the tumor with a nodule-in-nodule appearance, and the foci totally showed coagulative necrosis. On immunostaining, lymphocytes in the tumor stroma were positive for CD8 and programmed death 1. The expression of programmed death-ligand 1 was observed in carcinoma cells and macrophages specifically within the lymphocyte-rich area of HCC.

**Conclusions:**

We consider this case representative of spontaneous regression of HCC, and the immune response against HCC might contribute to tumor regression, leading to complex histopathological appearances. This case may provide insight into the mechanism of spontaneous regression of HCC.

## Background

Spontaneous regression of cancer is defined as a partial or complete disappearance of malignant tumors without treatment for prevention of tumor growth [1]. Spontaneous regression occurs in many types of tumors, such as renal cell carcinoma, neuroblastoma, malignant melanoma, choriocarcinoma, bladder carcinoma, leukemia, and breast cancer. Spontaneous regression of hepatocellular carcinoma (HCC) is a rare event with an incidence of only 0.4% [2]. There are several theories about the mechanism of spontaneous regression of HCC, such as impairment of blood supply and immune reaction, but no definitive mechanism has been established [3–7].

Herein, we present a rare case of HCC with unique histopathological features confirmed by surgical resection, which may represent the process of spontaneous regression of HCC. In this case, the tumor regression was not attributed to ischemia, and the immune response against HCC might have contributed to the regression as well as the complex histopathological appearances.

## Case presentation

An 84-year-old Japanese female with hepatitis C virus (HCV)-associated chronic hepatitis and diabetes mellitus treated at another hospital was referred to our hospital for further examination because of elevated α-fetoprotein (AFP) in a routine blood test. The patient was negative for a history of alcohol consumption, smoking, excessive weight loss and medications including herbs. The patient’s height and weight were 1.48 m and 45.4 kg, blood pressure was 138/76 mmHg, pulse rate was 70 beats/min and body temperature was 36.3 °C, respectively, with no specific physical abnormalities. The patient’s blood platelet count was found to be 16.0 × 10^4^/µl (normal range, 14.0–37.9 × 10^4^/µl) with slightly elevated aspartate aminotransferase levels of 35 IU/l (normal range, 13–30 IU/l) and alanine aminotransferase levels of 32 IU/l (normal range, 7–23 IU/l). The levels of AFP and protein induced by vitamin K absence or antagonist II (PIVKA-II) were both elevated, with values of 414 ng/ml (normal level, < 10 ng/ml) and 236 mAu/ml (normal level, < 40 mAu/ml), respectively. HbA1c was 7.4% (normal level, < 5.9%) under insulin treatment, and no improvement in diabetes mellitus was observed prior to admission. No inflammatory or coagulating system abnormalities were observed.

**Fig. 1:**
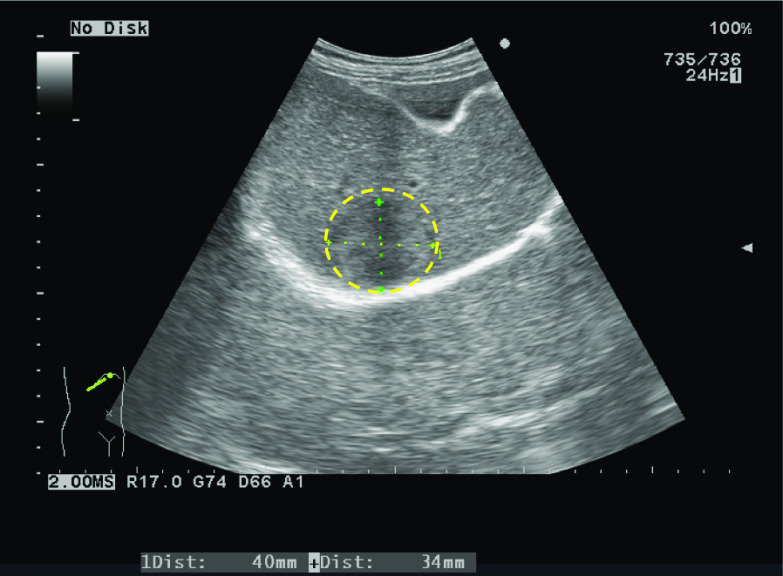
Abdominal ultrasonography (US). Abdominal US showed a 3-cm solid tumor in liver segment 8 with a heterogeneous irregular center and no fibrous capsule (dotted circle)

**Fig. 2:**
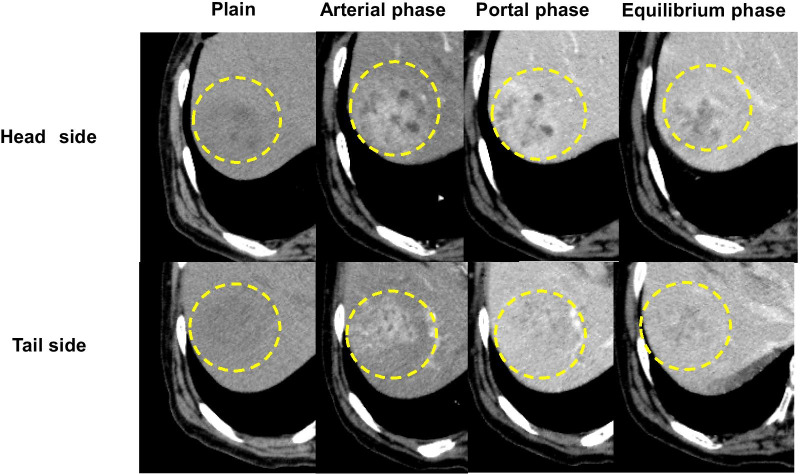
Abdominal contrast-enhanced computed tomography (CT) scan. An abdominal contrast-enhanced CT scan revealed a tumor 3 cm in size in liver segment 8 that showed heterogeneous enhancement in the arterial phase and washed out insufficiently in the portal and equilibrium phase (dotted circle)

**Fig. 3:**
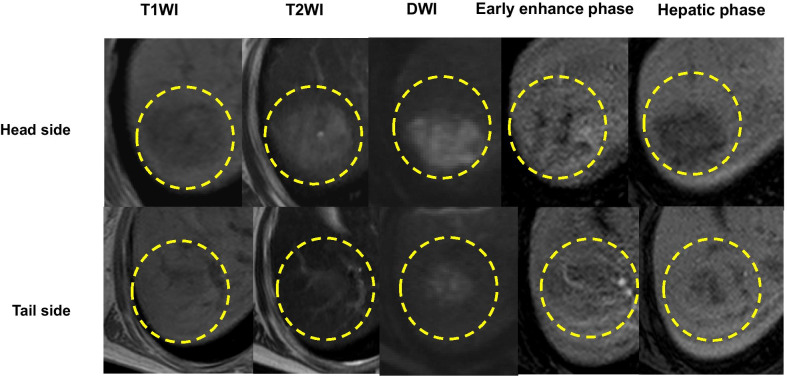
Magnetic resonance imaging (MRI). MRI revealed a tumor in liver segment 8 with low- to iso-intensity on T1-weighted imaging (T1WI), high- to iso-intensity on T2-weighted imaging (T2WI), high intensity on diffusion-weighted imaging (DWI) and low intensity on hepatocellular images, and the enhanced pattern was the same as that of computed tomography (dotted circle)

Abdominal ultrasonography showed a 3.4-cm solid tumor in liver segment 8 (S8), with a heterogeneous irregular center but no fibrous capsule (Fig. 1). A tumor 3 cm in size was observed in S8 on abdominal contrast-enhanced computed tomography (CT) performed at the time of admission, which showed heterogeneous enhancement in the arterial phase and washed out insufficiently in the portal and equilibrium phase (Fig. 2). Magnetic resonance imaging (MRI) revealed a tumor in S8 with low- to iso-intensity on T1-weighted imaging, high- to iso-intensity on T2-weighted imaging, high intensity on diffusion-weighted imaging and low intensity on hepatocellular imaging (Fig. 3), and the enhanced pattern was the same as that of the CT scan. As regards the enhanced pattern, HCC is always enhanced strongly in the arterial phase, and washed out in the following phase. On the contrary, in the present case, the tumor was enhanced heterogeneously in the arterial phase, and washed out insufficiently in the following phase. Although the imaging findings were not typical, HCC was the most probable diagnosis in this case. Therefore, liver resection (S8) was performed.

**Fig. 4:**
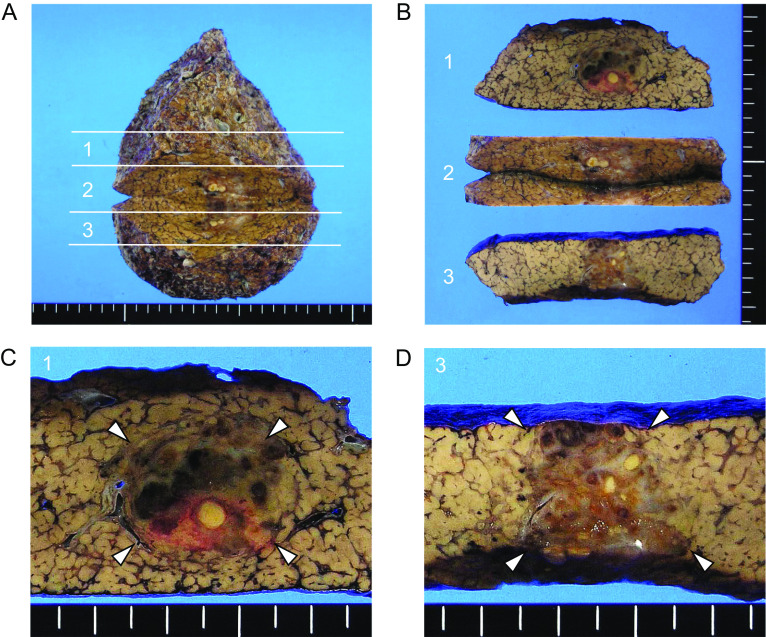
Gross appearance of surgically resected specimen. The resected specimen (**a**) was cut into thirds (**b**). The cut surface showed a tumor with a heterogeneous appearance (**c** and **d**, arrowheads) without fibrous capsule. The background liver was cirrhotic

**Fig. 5:**
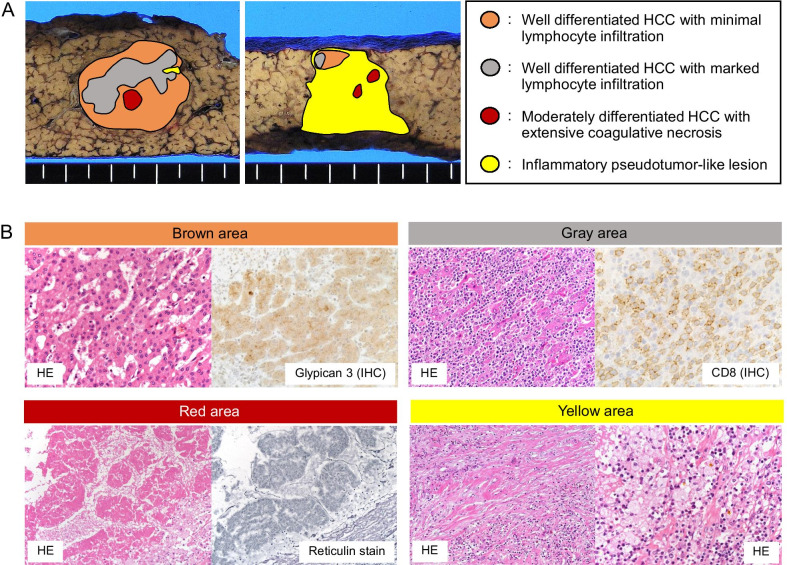
Illustrative presentation and histology of the tumor. The areas colored brown, gray, red and yellow were superimposed on the cut surface images of Fig. 1c, d. **a** Representative histological images corresponding to each colored area are shown in **b**. *IHC* immunohistochemistry

The surgically resected specimen contained a tumor 2.5 cm at the largest diameter without fibrous capsule. The cut surface of the tumor grossly showed a heterogeneous appearance (Fig. 4, arrowheads). Accordingly, histological examination showed that it had several different components. As illustrated in Fig. 5a, four major components could be identified, although the boundary between each was unclear.

In the nodular area colored brown in Fig. 5a, hepatocyte-like cells were arranged in a thin trabecular pattern with increased cellularity (Fig. 5b). This component was immunohistochemically positive for glypican3 and regarded as well-differentiated HCC. Within the nodule of well-differentiated HCC, an area accompanied by marked lymphocyte infiltration in the tumor stroma was noted (colored gray in Fig. 5a), and the tumor-infiltrating lymphocytes (TILs) were predominantly CD8^+^ T cells (Fig. 5b). Epstein–Barr virus infection was not detected in carcinoma cells by an in situ hybridization technique. Lymphocyte infiltration did not spread throughout the nodule of HCC, and the feature did not fulfill the criteria of HCC of lymphocyte-rich subtype according to the latest WHO classification of tumors of the digestive system, in which lymphocytes must outnumber tumor cells in most microscopic fields [8]. The area of well-differentiated HCC with or without TILs occupied approximately half of the entire tumor.

The other half of the tumor was replaced by fibrotic tissue accompanied by infiltration of lymphocytes and foamy macrophages, colored in yellow in Fig. 5a. The histologic picture of this area closely resembled inflammatory pseudotumor of the fibrohistiocytic type (Fig. 5b). Additionally, small nodular lesions that grossly showed pale yellow color were scattered within the nodule of well-differentiated HCC and inflammatory pseudotumor-like lesion (colored red in Fig. 5a). The small nodules were entirely composed of necrotic tissue, and a thick trabecular pattern could be recognized by reticulin staining (Fig. 5b), indicating moderately differentiated HCC with extensive coagulative necrosis, existing in a nodule-in-nodule pattern.

**Fig. 6:**
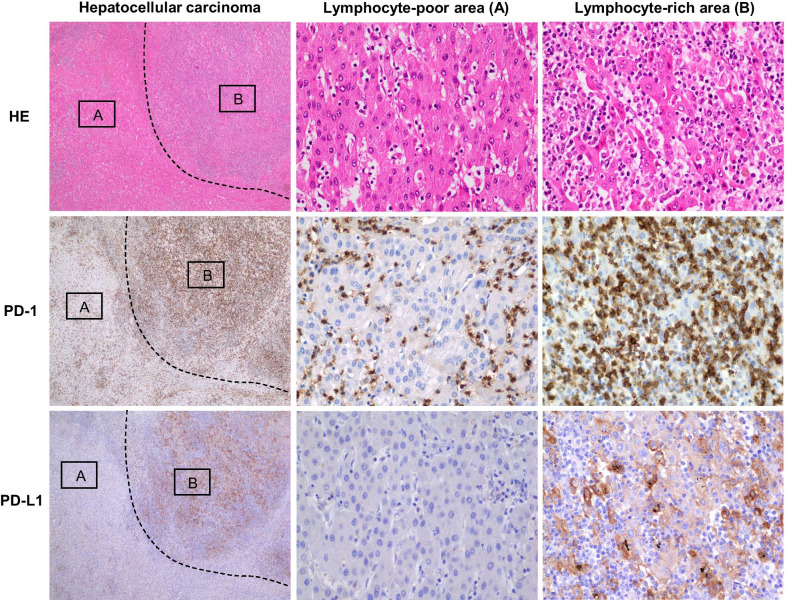
Immunohistochemical expression of programmed death 1 (PD-1) and programmed death-ligand 1 (PD-L1) in hepatocellular carcinoma (HCC). Lymphocytes infiltrating the tumor stroma were positive for PD-1. The expression of PD-L1 was observed in carcinoma cells and macrophages specifically within the lymphocyte-rich area of HCC

To address the status of tumor immunity, immunostaining of programmed death 1 (PD-1) and programmed death-ligand 1 (PD-L1) were performed. The expression of PD-1 was diffusely observed on TILs, and its expression was observed irrespective of the extent of infiltration of TILs within the tumor (Fig. 6). PD-L1 was expressed on carcinoma cells and tumor-associated macrophages (Fig. 6). Interestingly, the positivity of PD-L1 expression was restricted to the area of well-differentiated HCC with dense infiltration of TILs, and other areas of the tumor were negative for PD-L1 expression. The analysis of microsatellite instability was performed for five mononucleotide markers (BAT25, BAT26, NR21, NR24, and MONO27) using tumor and normal DNA obtained from formalin‐fixed, paraffin**-**embedded tissues. In the analysis, no unstable marker was detected, showing that the tumor was microsatellite-stable.

The background liver showed cirrhosis with lymphocyte infiltration in portal tracts, which was consistent with liver cirrhosis due to HCV infection. The histological changes related to nonalcoholic fatty liver diseases were unremarkable in the background liver.

The patient’s postoperative course was favorable, and she was discharged 27 days after surgery. The values of AFP and PIVKAII were both normalized. Since then, she has visited the hospital regularly for more than 6 years, with no signs of HCC recurrence on CT scan.

## Discussion

Spontaneous regression has been described in many types of tumors, especially renal cell carcinoma, melanoma, and neuroblastoma, which constitute nearly half of all reported cases of spontaneous tumor regression. In patients with HCC, the estimated incidence of spontaneous regression is 0.4% [2]. In the literature on spontaneous regression of HCC, several mechanisms of this phenomenon have been suggested. Possible mechanisms of tumor regression are divided into three major categories: (i) impairment of blood supply due to arterial thrombosis; (ii) immune reaction, and (iii) other factors, including abstinence from alcohol consumption, the use of herbal medicines, systemic inflammatory reactions, discontinuation of immunosuppressive therapy, sudden enlargement of the tumor, and reduced blood supply to cancer due to the formation of a fibrous capsule [3–7, 9–12].

In the present case, the following causes can be excluded: arterial thrombosis, abstinence from alcohol consumption, the use of herbal medicines or any other drugs, systemic inflammatory reactions, and the formation of a fibrous capsule. Although the precise mechanism of spontaneous regression of HCC could not be fully explained in the present case, the immune reaction might have played an important role because a large number of lymphocytes infiltrated the tumor stroma of HCC, and these cells were predominantly CD8^+^ T cells. Effective CD8^+^ T cells that mediate cytotoxic killing may play a crucial role in the antitumor immune reaction in the tumor microenvironment [13]. This character coincided with that in the present case. TILs, including T cells, B cells and natural killer cells, are representative components of the host antitumor immune response [13]. Increased numbers of TILs, particularly activated cytotoxic T cells, are reported to correlate with better survival in some malignant tumors, including HCC [14]. A meta-analysis revealed that high levels of intratumoral CD8^+^ TILs were associated with better overall survival [15]. However, in HCC patients, the prognostic role of TILs for survival remains controversial, with several studies reporting contradictory findings [16, 17].

From a therapeutic point of view, immunologically active tumors are predicted to respond to immunotherapy, which enhances the local antitumor immune response either through blocking coinhibitory checkpoint receptors or activating costimulatory receptors expressed on tumor-infiltrating T cells [18]. In the present case, PD-L1-positive cancer cells and macrophages were observed in the area of well-differentiated HCC with dense infiltration of PD-1-positive lymphocytes, and immune evasion via the PD-1/PD-L1 axis might be occurring in this area. It is speculated that PD-L1 positivity in HCC is a result of epigenetic events in which PD-L1 expression is induced in cancer cells in response to T cell infiltration, and this may be regarded as adaptive resistance of cancer cells to antitumor immunity. In such circumstances, blockade of the PD1/PD-L1 axis may represent a promising therapeutic option. In contrast, the areas of HCC lacking dense infiltration of TILs were negative for PD-L1 expression, and the immune response might be preserved in these areas.

In this case, CD8^+^ T cells probably injured HCC directly. As a result, coagulative necrosis of HCC and the formation of inflammatory pseudotumor-like lesions might be induced. We consider that the unique histopathological features represent one aspect of the time course of spontaneous regression of HCC, which accounts for the radiological image findings that were not typical for HCC. We did not perform liver biopsy for this tumor prior to surgery. However, our case strongly suggests that the presence of inflammatory pseudotumor-like lesions in biopsy specimens does not rule out the possibility of malignancy, including HCC, and careful interpretation is required.

## Conclusions

A case of possible spontaneous regression of HCC in a Japanese female patient was reported. The immune response against HCC might contribute to tumor regression, leading to complex histopathological appearances such as dense infiltration of TILs, coagulative necrosis of HCC, uneven expression of PD-L1 within the tumor, and the formation of inflammatory pseudotumor-like lesions. Cases of HCC showing such features have not been described in the literature, and this case may provide initial insights into the mechanism of the spontaneous regression of HCC.

## Data Availability

All data generated or analyzed during this study are included in this published article.

## Abbreviations

AFP: α-Fetoprotein
CT: Computed tomography
DWI: Diffusion-weighted imaging
HCV: Hepatitis C virus
IHC: Immunohistochemistry
MRI: Magnetic resonance imaging
PIVKA-II: Protein induced by vitamin K absence or antagonist II
PD-1: Programmed death 1
PD-L1: Programmed death-ligand 1
TIL: Tumor-infiltrating lymphocyte
US: Ultrasonography
